# Analysis of codon usage bias of thioredoxin in apicomplexan protozoa

**DOI:** 10.1186/s13071-023-06002-w

**Published:** 2023-11-21

**Authors:** Dawei Wang, Baoling Yang

**Affiliations:** Jinzhou Medical University, Jinzhou, 121000 Liaoning Province China

**Keywords:** Apicomplexan Protozoa, Thioredoxin, Codon usage bias, Relative synonymous codon usage, Effective number of codons

## Abstract

**Background:**

Apicomplexan protozoa are a diverse group of obligate intracellular parasites causing many diseases that affect humans and animals, such as malaria, toxoplasmosis, and cryptosporidiosis. Apicomplexan protozoa possess unique thioredoxins (Trxs) that have been shown to regulate various cellular processes including metabolic redox regulation, parasite survival, and host immune evasion. However, it is still unknown how synonymous codons are used by apicomplexan protozoa Trxs.

**Methods:**

Codon usage bias (CUB) is the unequal usage of synonymous codons during translation which leads to the over- or underrepresentation of certain nucleotide patterns. This imbalance in CUB can impact a variety of cellular processes including protein expression levels and genetic variation. This study analyzed the CUB of 32 Trx coding sequences (CDS) from 11 apicomplexan protozoa.

**Results:**

The results showed that both codon base composition and relative synonymous codon usage (RSCU) analysis revealed that AT-ended codons were more frequently used in *Cryptosporidium* spp. and *Plasmodium* spp., while the *Eimeria* spp., *Babesia* spp., *Hammondia hammondi*, *Neospora caninum*, and *Toxoplasma gondii* tended to end in G/C. The average effective number of codon (ENC) value of these apicomplexan protozoa is 46.59, which is > 35, indicating a weak codon preference among apicomplexan protozoa Trxs. Furthermore, the correlation analysis among codon base composition (GC1, GC2, GC3, GCs), codon adaptation index (CAI), codon bias index (CBI), frequency of optimal codons (FOP), ENC, general average hydropathicity (GRAVY), aromaticity (AROMO), length of synonymous codons (L_sym), and length of amino acids (L_aa) indicated the influence of base composition and codon usage indices on CUB. Additionally, the neutrality plot analysis, PR2-bias plot analysis, and ENC-GC3 plot analysis further demonstrated that natural selection plays an important role in apicomplexan protozoa Trxs codon bias.

**Conclusions:**

In conclusion, this study increased the understanding of codon usage characteristics and genetic evolution of apicomplexan protozoa Trxs, which expanded new ideas for vaccine and drug research.

**Graphical Abstract:**

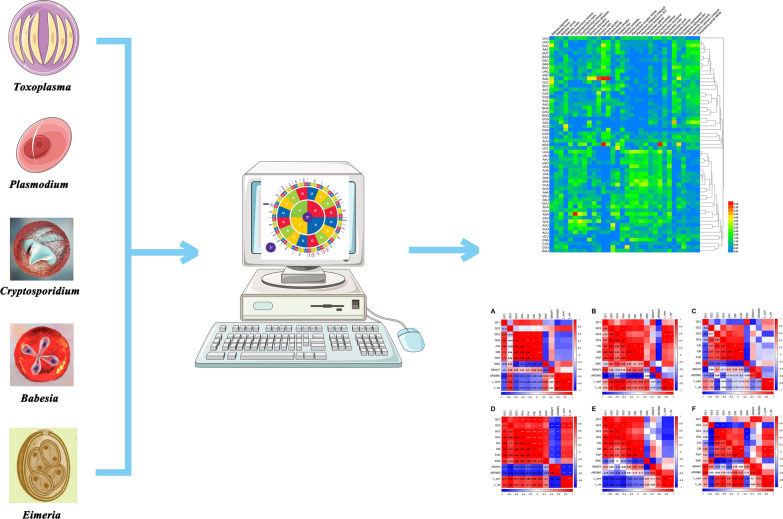

**Supplementary Information:**

The online version contains supplementary material available at 10.1186/s13071-023-06002-w.

## Background

Proteins serve as the primary agents responsible for biological functions and are primarily comprised of 20 standard amino acids. The 20 standard amino acids are denoted by 64 codons, out of which 61 encode amino acids, while the remaining three represent translation stop signals. With the exception of methionine (Met) and tryptophan (Trp), which are represented by a solitary codon, most species employ various synonymous codons to encode the remaining 18 amino acids [1–3]. Despite undergoing evolution over time, the genetic code remains highly conserved and permits the use of diverse codons or synonymous codons for encoding the same amino acid [4–6]. The frequency of synonymous codon usage is non-uniform and often random across various organisms, genes, or even the same gene among different species. In many cases, some codons are favored over others for amino acid encoding purposes [7–9]. Codon usage bias (CUB) is a prevalent occurrence wherein synonymous codons manifest with distinct frequencies [10–13]. Throughout the course of evolution, synonymous mutations, also known as “silent mutations,” are not anticipated to modify the original sequence or primary structure of proteins. Therefore, variations in synonymous codons among organisms can significantly contribute to genome evolution [14]. Many previous studies have noted that multiple factors affect CUB in different organisms, of which the basic factors for CUB are assumed to be a balance between natural selection (e.g. translational selection, gene length, and gene function) and mutation bias (such as GC content and mutation position of base) as well as the influence of random genetic drift [14–18]. CUB is known to have a significant impact on a wide range of cellular processes such as mRNA stability, transcription, translation efficiency and accuracy, as well as protein structure, folding, expression, and function. Additionally, there are various significant practical applications for understanding CUB, including heterologous gene expression [19], identifying species origins [6, 20], designing degenerate primers [21], predicting gene expression levels [22, 23], predicting gene functions [24, 25], and designing synthetic genes for biotechnological applications [26]. However, most of the numerous studies on CUB have focused on bacterium, fungi, viruses, and mycoplasma [27–31]. Thus far, the genetic features of codon bias in parasites, particularly in apicomplexan protozoa, have not been comprehensively comprehended.

Apicomplexans are a diverse group of protozoa that are obligate intracellular parasites and are responsible for causing many diseases that affect humans and animals, including *Toxoplasma gondii*, *Neospora caninum*, *Plasmodium* spp., *Cryptosporidium* spp., *Eimeria* spp., *Babesia* spp., *Theileria* spp. [32–38]. They have a complex life cycle involving multiple hosts and typically have an apical complex that aids in penetrating host cells. Their cell structure includes a complex organelle called the apicoplast, which is derived from secondary endosymbiosis and is essential for parasite survival. Apicomplexans have been a topic of research due to their unique features, pathogenicity, and impact on global health. Multiple studies imply the invasion process of apicomplexans is mediated by many invasion-related protein molecules, including microneme proteins, rhoptry proteins, dense granule proteins, surface antigen proteins [39–44]. In recent years, many studies have shown that thioredoxin (Trx) is also involved in the invasion process of apicomplexan protozoa. Trx is a redox enzyme that regulates cellular redox homeostasis by catalyzing the reduction of disulfide bonds in proteins. Apicomplexan protozoa possess unique Trxs that have been shown to regulate various cellular processes including metabolic redox regulation, parasite survival, and host immune evasion in *T. gondii*, *Plasmodium falciparum*, *N. caninum*, *Babesia* spp., and *Cryptosporidium* spp. [45–51]. The Trx systems in apicomplexan protozoa have been identified as potential targets for the development of novel antiparasitic drugs. The functional domain of Trx in apicomplexan protozoa is conserved, but the coding sequences are vastly different, and research on apicomplexan protozoa Trx codon usage is rare. In this study, we systematically analyzed and compared the CUB of Trxs of 32 sequences from 11 apicomplexan protozoa, including *Babesia* spp., *Besnoitia besnoiti*, *Cryptosporidium* spp., *Cyclospora cayetanensis*, *Eimeria* spp., *Gregarina niphandrodes*, *Hammondia hammondi*, *N. caninum* Liverpool, *Plasmodium* spp., *Theileria* spp., and *T. gondii*. The phylogenetic tree of apicomplexan protozoa was constructed based on the relative synonymous codon usage of Trxs, which was compared with the phylogenetic tree constructed according to the Trx coding sequences (CDSs). Therefore, analyzing the CUB can provide further information on the genetics and evolution of species and help accurately predict the function and expression regulation mechanisms of related genes.

## Methods

### Sequences

A total of 32 Trxs complete coding sequences from 11 apicomplexan protozoa were retrieved from the National Center for Biotechnology Information (NCBI) GenBank database (https://www.ncbi.nlm.nih.gov/genbank/) for subsequent CUB analysis. Detailed information about the overall 32 Trx CDS is listed in Additional file 1: Table S1.

### Analysis of codon base composition

In this study, CodonW software was used to determine the contents of the nucleotide at the third codon location (C3, T3, G3, and A3%) for all synonymous codons in apicomplexan protozoa Trxs. Furthermore, the GC% contents of all three codon locations (GC1, GC2, and GC3%) and total GCs% and ATs% contents were measured. Only 59 synonymous codons encoding 18 amino acids were considered for the present study, not including the first AUG codon (Met), the codon (UGG) encoding Trp, and the three termination codons (UAG, UAA, and UGA), respectively [52].

### Analysis of codon usage indices

Mutational pressure and natural selection are two key factors for codon bias. For this, many statistical methods have been proposed to analyze the codon usage indices and then determine which one is the driving force in this study. The codon adaptation index (CAI) is applied to calculate the gene expression level depending on its codon-based sequence through an online tool used for CAI calculation. It ranges from zero to one; the larger the value is, the more frequent the CUB. Thus, CAI is useful for predicting the expression level of a particular gene [53]. The codon bias index (CBI) is used as a standard to evaluate gene expression, which reflects the components of highly expressed superior codons in a specific gene [54]. The frequency of optimal codons (FOP) is calculated by counting the ratio of the optimal codon number to the total synonymous codon number in one specific gene. The FOP value varies and ranges from 0.36 (which means the codon usage bias is weak) to 1 (which means the codon usage bias is strong). The value of CBI near zero indicates all codons are completely randomly used [55]. The effective number of codons (ENC) refers to the number of effective codons used in one specific gene. The ENC value varies and ranges from 20 (which means that only one codon is used for each amino acid) to 61 (which means that each codon is used on average). In addition, if the value of ENC is < 35, the codon usage bias is strong; if it is > 35, the codon is randomly used [56]. The general average hydropathicity (GRAVY) values were calculated by the arithmetic mean of the sum of the hydropathic indices of each amino acid. GRAVY values range from − 2 to 2; positive and negative values represent hydrophobic and hydrophilic proteins, respectively [57]. The aromaticity (AROMO) value represents the frequency of aromatic amino acids (Phe, Tyr, and Trp) in a specific gene [58]. The length of synonymous codons (L_sym) and length of amino acids (L_aa) are the two indices which represent the number of synonymous codons and the number of translatable codons, respectively. The variation in amino acid composition can also influence the analysis results of codon usage [59].

### Analysis of relative synonymous codon usage

Relative synonymous codon usage (RSCU) value was calculated by dividing the amino acids encoded by the same codons and their probability of appearing in the same codons. An RSCU value > 1 indicates a positive codon bias (RSCU value > 1.6 indicates a strong positive codon bias), an RSCU value < 1 indicates a negative codon bias, and an RSCU value = 1 indicates a random codon usage [60].

### Neutrality plot analysis

The neutrality plot can explain the balance between mutation pressure and natural selection in specific genes. The line of regression slope between GC3 and GC12 (the average GC codon content in GC1 and GC2) indicates that mutation pressure is the major factor affecting CUB when values come close to 1. In contrast, if there is no correlation between GC12 and GC3, the value comes close to 0, and then the main driving force of the tested gene is natural selection [61].

### PR2-bias plot analysis

Parity Rule 2 bias (PR2-Bias) plot analyses were performed based on [A3/(A3 + U3) vs. G3/(G3 + C3)]. If the codon had no usage bias, A = T and C = G, the value was in the center point of the plot. In contrast, the other vectors emitted from the center point indicate the degree and direction of the gene bias [62].

### ENC-GC3 plot analysis

The ENC-GC3 plot (ENC vs. GC3) is usually used to analyze the influencing factor of CUB in a specific gene, such as mutation pressure and natural selection. The ENC-GC3 diagram consists of the ordinate ENC value and abscissa GC3 value, and the standard curve shows the functional relation between ENC and GC3. If the corresponding points are distributed around or on the standard curve, we can conclude that the mutation pressure is an independent force in CUB. If the corresponding point is lower or far from the standard curve, the natural selection factor may play a key role in the formation of codon bias [63].

### Correlation analysis

Correlation analysis was performed to illustrate the relationship among codon base composition (GC1, GC2, GC3, GCs), CAI, CBI, FOP, ENC, GRAVY, AROMO, L_sym, and L_aa of apicomplexan protozoa Trxs. Spearman’s rank correlation method was applied in correlation analysis. All processes were executed using the R corrplot package [64].

### Phylogenetic analysis

The clustering analysis to the RSCU of Trxs was made among 32 representative apicomplexan protozoa using the method of squared Euclidean distance [2]. The phylogenetic tree was constructed using the neighbor-joining method by MEGA 11.0 (https://www.megasoftware.net/), and a cluster heat map was generated by Hemi 1.0 software (http://hemi.biocuckoo.org/down.php).

### Software used

All indices of codon usage bias above were calculated in the data set using the program CodonW 1.4.2 (http://codonw.sourceforge.net/). Clustering and correlation analyses were conducted using the statistical software SPSS 18.0. Graphs were generated in GraphPad Prism 6.01 (http://www.graphpad.com/scientific-software/prism/).

## Results

### Results of codon base composition in apicomplexan protozoa Trxs

**Fig. 1:**
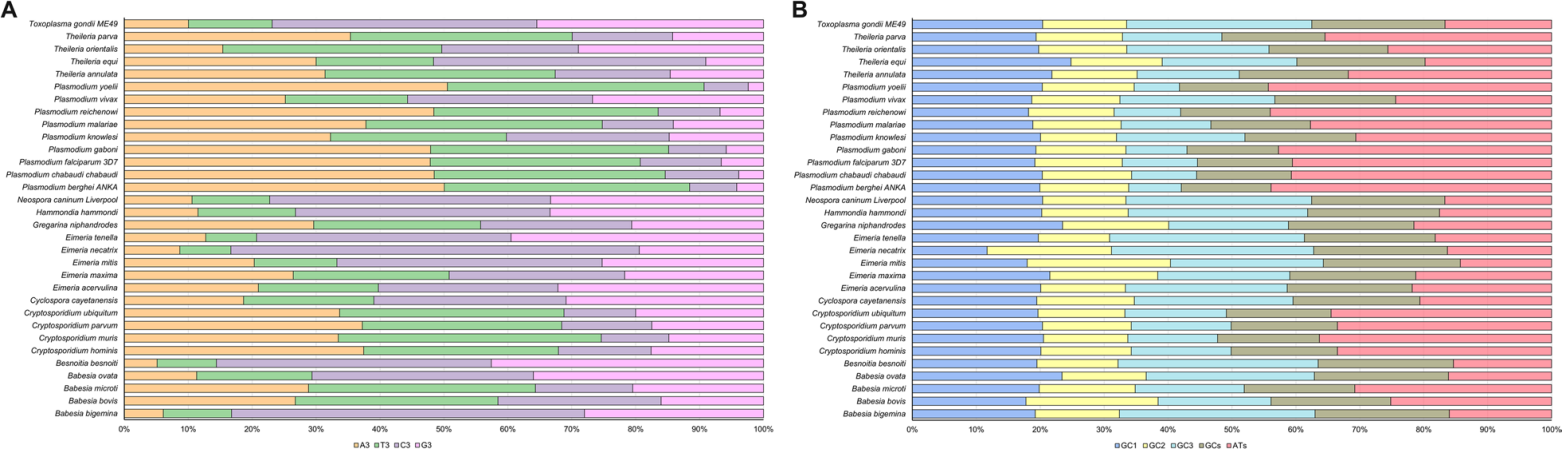
Codon base composition in apicomplexan protozoa Trxs. The relative content of codon base composition in apicomplexan protozoa Trxs were calculated. **A** List the contents of the nucleotide at the third codon location (C3, T3, G3, and A3%) in apicomplexan protozoa Trxs. **B** GC% contents of all three codon locations (GC1, GC2, and GC3%) and total GCs% and ATs% contents in apicomplexan protozoa Trxs. X-axis represents percentages, while Y-axis represents different apicomplexan protozoa

CUB can be considerably influenced by the general base composition of genomes. We selected 32 Trxs from the 11 apicomplexan protozoa for codon usage analysis (Additional file 1: Table S1). Statistical analysis found that the encoding region length of these Trx ranged from 255 to 1665 bp, with the *Plasmodium vivax* Trx gene having the longest length and the *Eimeria necatrix* Trx gene having the shortest. We further calculated the base composition of 32 Trxs, and our outcomes disclosed that *Plasmodium* spp. and *Cryptosporidium* spp. are rich in the A3, T3, and ATs bases, and *Eimeria* spp. and *Babesia* spp. are rich in the G3, C3, and GCs bases (Fig. 1, Additional file 2: Table S2). The content of T3% is most in *Cryptosporidium muris* (56.52%) and least in *E. necatrix* (9.86%). The A3% content of *Plasmodium yoelii* (69.15%) is at a maximum level higher than that in other apicomplexan protozoa, while the content of G3% (3.27%) and C3% (9.46%) in *P. yoelii* is least among these apicomplexan protozoa (Fig. 1A, Additional file 2: Table S2). In addition, nucleotide content analysis at the first, second, and third synonymous codon positions showed that the values of GC1% ranged from 31.31 to 63.12% (mean: 44.52%), while GC3% ranged from 12.11 to 85.78% (mean: 46.98%). However, the GC2% values ranged from 23.1 to 62.75%; the average value is the lowest (mean: 32.57%, Fig. 1B, Additional file 2: Table S2).

### Results of codon usage index analysis in apicomplexan protozoa Trxs

We calculated the CAI values of 32 Trxs from the 11 apicomplexan protozoa and found that the CAI values of Trxs ranged from 0.171 to 0.373 (Table 1). Among them, *C. muris* had the lowest CAI value, while the *E. necatrix* had the highest CAI value, indicating that the *E. necatrix* gene had a high codon bias. In terms of species, the Trxs of *Eimeria* spp. has the highest CAI value, followed by *H. hammondi*, while the CAI values of *Cryptosporidium* spp. are the lowest, indicating that *Eimeria* spp. have strong codon bias. The CBI values of the 32 Trxs that were detected ranged from − 0.209 to 0.415 (Table 1). Among them, *Cryptosporidium ubiquitum* had the lowest CBI value, and *E. necatrix* had the highest, which has a strong codon bias. The average FOP values ranged from 0.305 to 0.679 among the 32 Trxs detected, while *C. muris* had the lowest FOP value and *E. necatrix* had the highest FOP value with strong codon bias (Table 1). We further calculated the GRAVY values of 32 Trxs and the results showed that 25 of the 32 Trxs had negative GRAVY values, indicating that they might be hydrophilic proteins, while most *Theileria* spp. were considered hydrophobic (Table 1). The frequency of aromatic amino acids (AROMO value) ranges from 0.049 to 0.153 (Table 1). *Babesia bovis* has the highest AROMO value, while *Eimeria mitis* is the lowest. The AROMO values of different apicomplexan protozoa Trxs varied obviously, with an average of 0.103. The average ENC value of all 32 Trxs detected ranged from 30.77 to 61, with an average ENC value of 46.59. Only the ENC value of *E. necatrix* was < 35, and the others were more than 35, even equal to 61, indicating that these genes had a weak codon usage preference (Table 1). The data of L_sym (range from 81 to 534) and L_aa (range from 85 to 555) are listed in Table 1.

**Table 1:** Codon usage indices in apicomplexan protozoa Trxs

Species	CAI	CBI	FOP	ENC	GRAVY	AROMO	L_sym	L_aa
*Babesia bigemina*	0.305	0.177	0.525	35.62	0.291	0.107	99	103
*Babesia bovis*	0.208	− 0.030	0.408	59.98	0.074	0.153	360	372
*Babesia microti*	0.174	− 0.151	0.330	58.81	− 0.222	0.117	182	188
*Babesia ovata*	0.287	0.105	0.482	54.59	− 0.334	0.093	137	140
*Besnoitia besnoiti*	0.284	0.137	0.496	36.15	− 0.192	0.098	411	428
*Cryptosporidium hominis*	0.178	− 0.100	0.364	51.18	− 0.321	0.070	198	201
*Cryptosporidium muris*	0.171	− 0.206	0.305	41.70	− 0.325	0.081	177	185
*Cryptosporidium parvum*	0.176	− 0.130	0.345	44.25	− 0.322	0.070	197	201
*Cryptosporidium ubiquitum*	0.172	− 0.209	0.308	50.70	− 0.344	0.085	195	201
*Cyclospora cayetanensis*	0.271	− 0.022	0.420	50.21	− 0.384	0.076	100	105
*Eimeria acervulina*	0.241	− 0.066	0.385	50.81	− 0.067	0.098	96	102
*Eimeria maxima*	0.233	0.056	0.460	48.49	− 0.248	0.097	420	433
*Eimeria mitis*	0.327	0.275	0.599	42.28	− 0.410	0.049	299	305
*Eimeria necatrix*	0.373	0.415	0.679	30.77	0.225	0.106	81	85
*Eimeria tenella*	0.315	0.177	0.520	48.72	− 0.353	0.087	98	103
*Gregarina niphandrodes*	0.207	0.052	0.435	61.00	0.026	0.107	193	197
*Hammondia hammondi*	0.307	0.195	0.531	46.08	− 0.202	0.104	409	423
*Neospora caninum Liverpool*	0.276	0.142	0.496	45.32	− 0.249	0.093	415	428
*Plasmodium berghei* ANKA	0.179	− 0.133	0.350	35.82	− 0.348	0.121	406	420
*Plasmodium chabaudi chabaudi*	0.200	− 0.067	0.389	39.47	− 0.342	0.124	406	420
*Plasmodium falciparum* 3D7	0.192	− 0.118	0.356	42.84	− 0.301	0.123	413	424
*Plasmodium gaboni*	0.174	− 0.153	0.337	39.70	− 0.308	0.125	413	424
*Plasmodium knowlesi*	0.246	0.027	0.436	59.94	− 0.317	0.119	413	427
*Plasmodium malariae*	0.188	− 0.112	0.357	51.86	− 0.269	0.124	406	420
*Plasmodium reichenowi*	0.174	− 0.191	0.314	38.19	− 0.255	0.125	322	329
*Plasmodium vivax*	0.251	0.102	0.489	54.70	− 0.624	0.105	534	555
*Plasmodium yoelii*	0.178	− 0.134	0.349	35.87	− 0.370	0.121	407	420
*Theileria annulata*	0.185	− 0.101	0.342	45.83	0.173	0.094	146	149
*Theileria equi*	0.255	0.053	0.447	37.17	0.274	0.105	103	105
*Theileria orientalis*	0.203	− 0.115	0.348	59.42	− 0.168	0.079	135	140
*Theileria parva*	0.195	− 0.091	0.359	47.64	0.212	0.134	220	224
*Toxoplasma gondii* ME49	0.293	0.162	0.511	45.70	− 0.187	0.101	409	424

CAI, codon adaptation index; CBI, codon bias index; FOP, frequency of optimal codons; ENC, effective number of codons; GRAVY, general average hydropathicity; AROMO, aromaticity; L_sym, length of synonymous codons; L_aa, length of amino acids were calculated in apicomplexan protozoa Trxs by Codon W software, respectively

Single underline represents the minimum value; double underline represents maximum value

### Defining codon usage patterns in apicomplexan protozoa Trxs

**Fig. 2:**
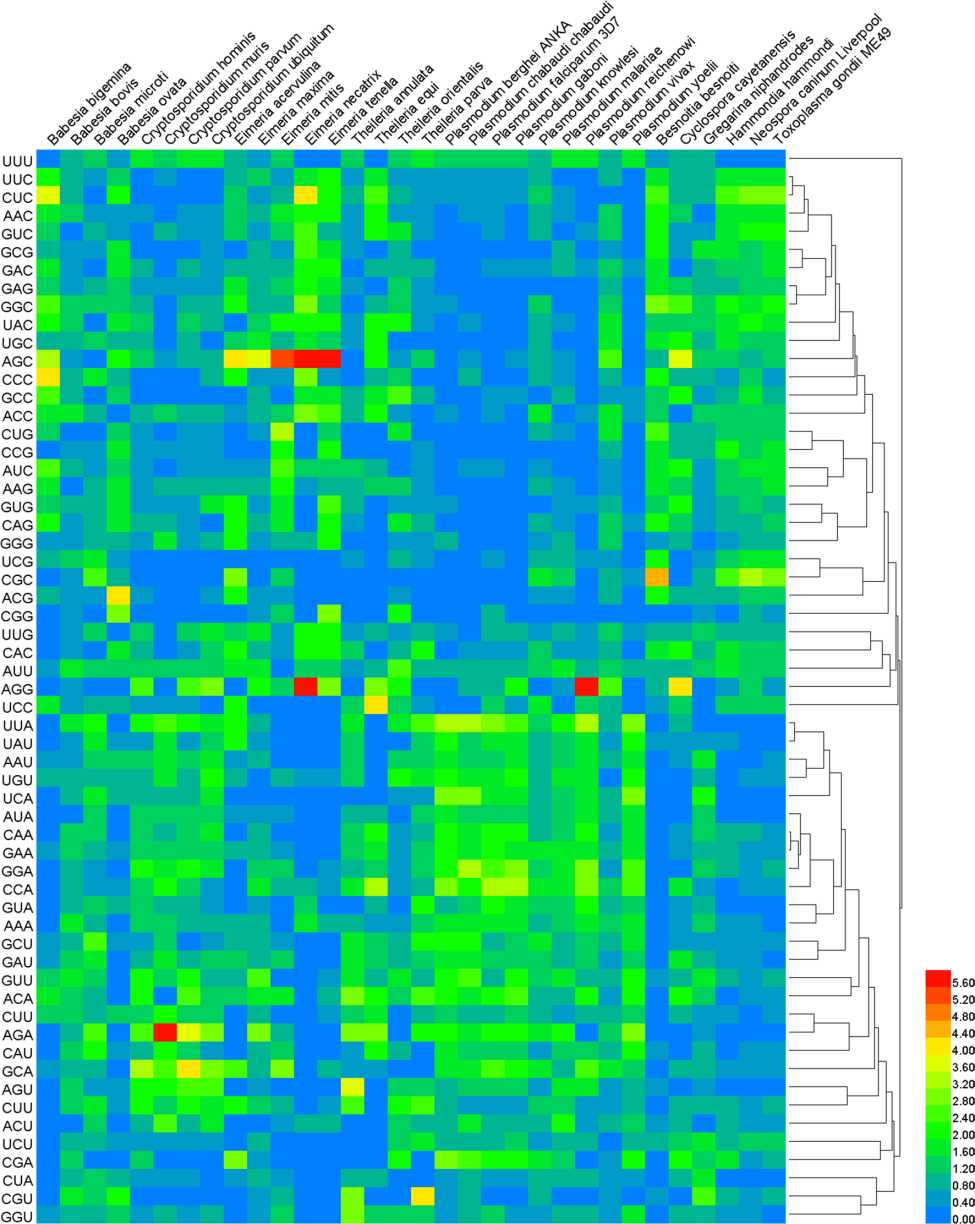
RSCU values in apicomplexan protozoa Trxs. Relative synonymous codon usage (RSCU) value was calculated by dividing the amino acids encoded by the same codons and their probability of appearing in the same codons. The color of the color block changes from blue to red, indicating that the RSCU values are increasing, of which an RSCU value > 1 indicates a positive codon bias. The homology of codons are also shown on the right side of the figure

An RSCU analysis was used to regulate the identical pattern of codon usage in the Trxs of apicomplexan protozoa. CUB was found to occur among these parasites, and 31 of the 32 apicomplexan protozoa contained > 24 positive codon bias (RSCU ≥ 1), except *Babesia bigemina* (including 23 positive codon bias, Fig. 2, Additional file 3: Table S3). In addition, > 6 high-frequency codons (RSCU ≥ 1.6) among the 32 apicomplexan protozoa with 19 high-frequency codons in *Plasmodium berghei* ANKA, *P. yoelii*, and *B. besnoiti* indicate *Plasmodium* spp. have a stronger positive codon bias and only six high-frequency codons in *B. bovis*. Furthermore, from the RSCU analysis, we found that the most abundantly used codons in 32 apicomplexan protozoa are AGC (Ser) and UUA (Leu), while CGG (Arg) is seldom used, even never used in *Cryptosporidium* spp., *B. besnoiti*, *C. cayetanensis*, and *H. hammondi*. Among the optimal codons, the AGA (Arg), AGC (Ser), and AGG (Arg) have the highest value (RSCU = 6), followed by AGC (Ser, RSCU = 5.36) and CGC (Arg, RSCU = 4.5), indicating the strongest positive codon bias, while AAG (Lys) has the lowest value (RSCU = 0.04) among the 59 synonymous codons. In addition, GCA (Ala) is used as the optimal codon in *Cryptosporidium* spp., AGC (Ser) is used as the optimal codon in *Eimeria* spp., and CGC (Arg) is used as the optimal codon in *B. besnoiti*, *H. hammondi*, *N. caninum*, and *T. gondii*.

### Results of neutrality plot analysis in apicomplexan protozoa Trxs

**Fig. 3:**
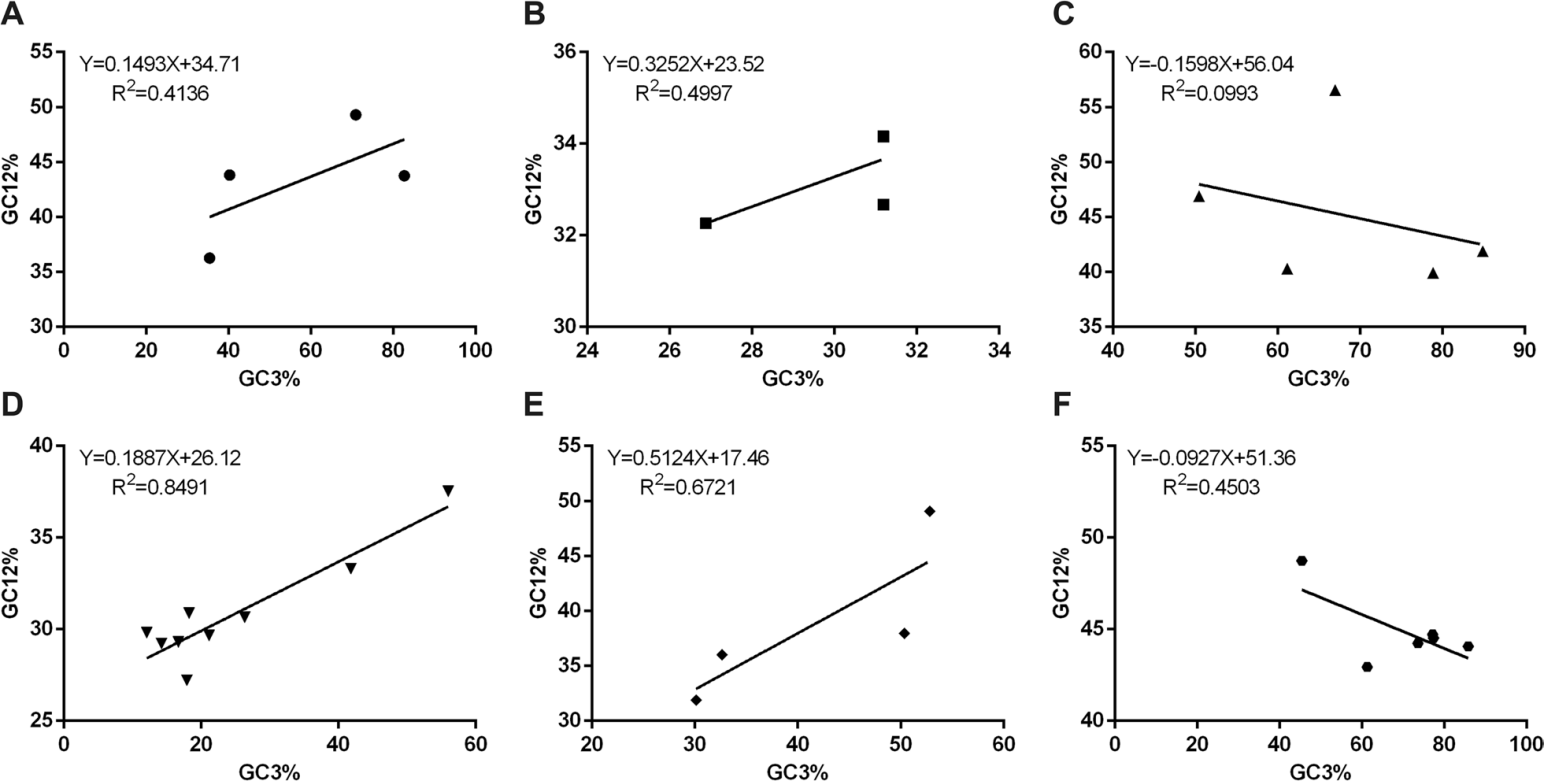
Neutrality plot analysis in apicomplexan protozoa Trxs. The correlations between the average GC codon content in GC1 and GC2 (GC12) and the third codon position (GC3) were analyzed and the standard curve and R^2^ in apicomplexan protozoa Trxs, respectively. X-axis represents GC3%, while Y-axis represents GC12%. **A** *Babesia* spp., **B** *Cryptosporidium* spp., **C** *Eimeria* spp., **D** *Plasmodium* spp., **E** *Theileria* spp., **F** others (including *Besnoitia besnoiti*, *Cyclospora cayetanensis*, *Gregarina niphandrodes*, *Hammondia hammondi*, *Neospora caninum*, and *Toxoplasma gondii*)

A plot of neutrality was performed, which implied the relationships between GC12 and GC3 composition to determine the position of mutation pressure and natural selection that has an impact on the CUB form. The GC12 content varied from 27.21 to 56.54%, and the GC3 content varied from 12.11 to 85.78% (Additional file 2: Table S2). To observe the association, we programmed a paradigm on the plot of neutrality between GC12 and GC3 for the 32 Trxs in apicomplexan protozoa. These 32 apicomplexan protozoa were divided into six groups: (A) *Babesia* spp., (B) *Cryptosporidium* spp., (C) *Eimeria* spp., (D) *Plasmodium* spp., (E) *Theileria* spp., and (F) others (including *B. besnoiti*, *C. cayetanensis*, *G. niphandrodes*, *H. hammondi*, *N. caninum*, and *T. gondii*). The slopes of the regression lines ranged from − 0.1598 (*Eimeria* spp.) to 0.5124 (*Theileria* spp.), indicating that the content of GC12 and GC3 in apicomplexan protozoa Trxs is weakly associated (Fig. 3). In addition, the *R*^2^ value of the standard curve ranged from 0.993 (*Eimeria* spp.) to 0.8491 (*Plasmodium* spp.). There was no significant correlation between GC12 value and GC3 value (*p* > 0.05), which indicated that natural selection may play an important role in driving the evolution of Trxs in apicomplexan protozoa. This phenomenon is similar to the findings of previous studies.

### Results of PR2-bias plot analysis in apicomplexan protozoa Trxs

**Fig. 4:**
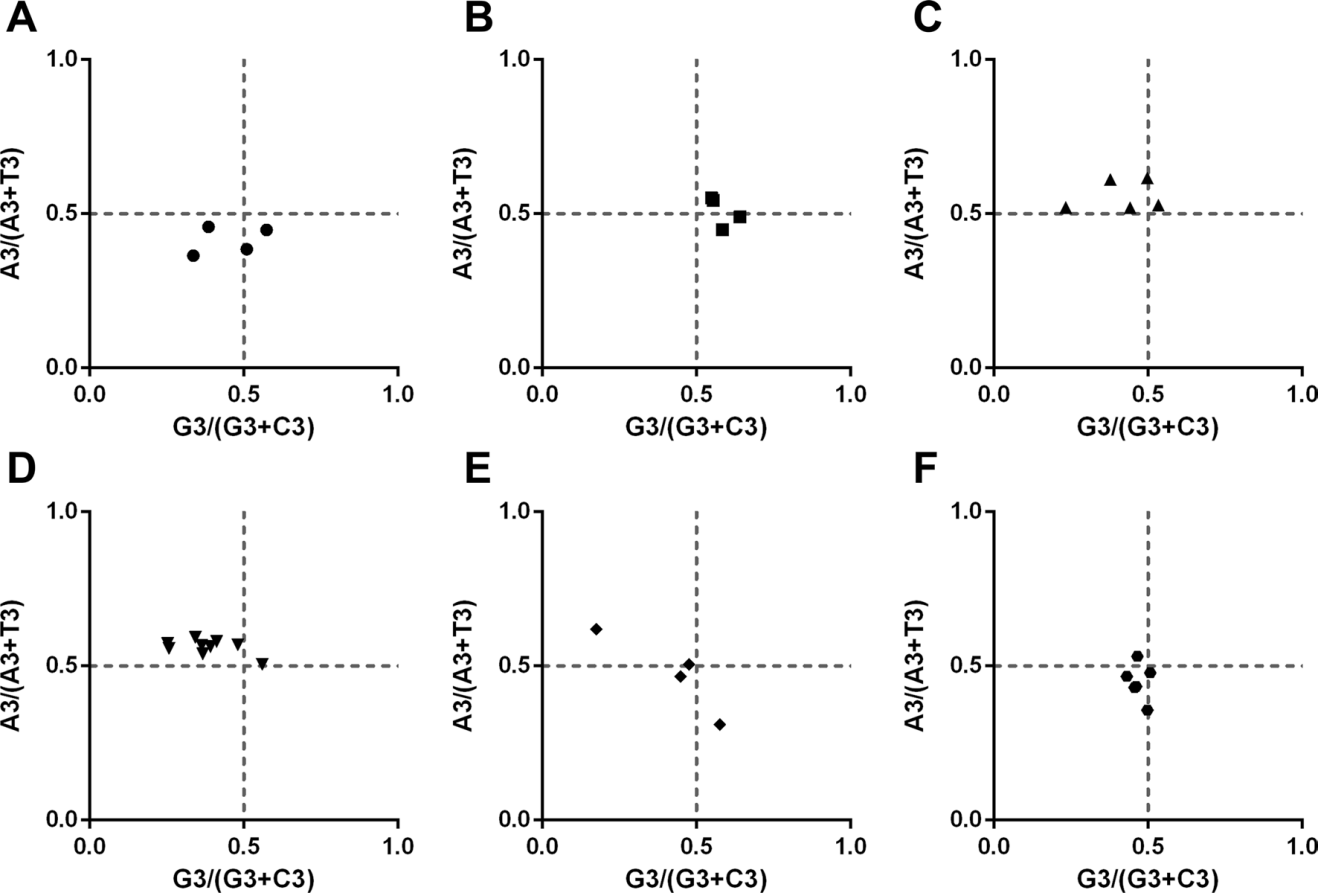
PR2-bias plot analysis in apicomplexan protozoa Trxs. The correlations between A3/(A3 + U3) and G3/(G3 + C3) were analyzed in apicomplexan protozoa Trxs, respectively. If the codon has no usage bias, A = T and C = G, the value was in the center point of the plot. The first quadrant represents the codon preference of A/G, and the third quadrant represents T/C preference. X-axis represents GC3%, while Y-axis represents GC12%. **A** *Babesia* spp., **B** *Cryptosporidium* spp., **C** *Eimeria* spp., **D** *Plasmodium* spp., **E** *Theileria* spp., **F** others (including *Besnoitia besnoiti*, *Cyclospora cayetanensis*, *Gregarina niphandrodes*, *Hammondia hammondi*, *Neospora caninum*, and *Toxoplasma gondii*)

To determine whether Trxs in apicomplexan protozoa have biases, we further performed a Parity Rule 2 (PR2) plot analysis (Fig. 4). Both axes were centered on 0.5 to divide the plot into four quadrants. In the first quadrant, the optimal codons are A and G, and the optimal codons are T and C in the third quadrant. The *Babesia* spp., *B. besnoiti*, *C. cayetanensis*, *H. hammondi*, *N. caninum*, and *T. gondii* prefer codon T to A (Fig. 4A, F). The optimal codon in *Cryptosporidium* spp. is G (Fig. 4B). Most of the dots were found to be distributed in the second quadrant of the *Eimeria* spp. and *Plasmodium* spp. (preferring A to T and C to G, Fig. 4C, D), with random codon usage in *Theileria* spp. (Fig. 4E). The analysis results showed that other factors, such as natural selection, play an important role in the process of codon bias in apicomplexan protozoa.

### Results of ENC-GC3 plot analysis in apicomplexan protozoa Trxs

**Fig. 5:**
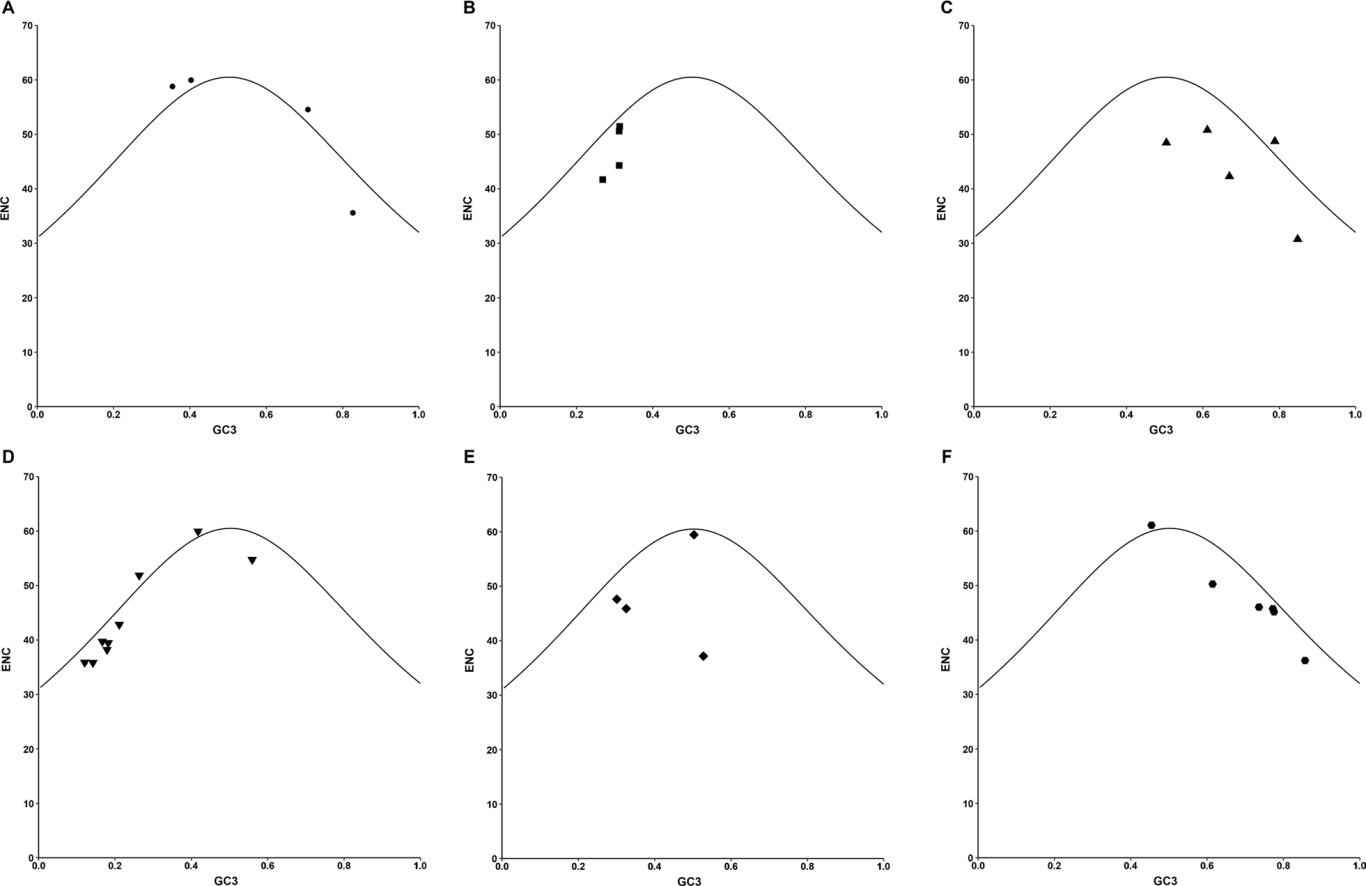
ENC-GC3 plot analysis in apicomplexan protozoa Trxs. The correlations between the effective number of codons (ENC) and the contents of the nucleotide G/C at the third codon location (GC3) were analyzed in apicomplexan protozoa Trxs, respectively. The standard curve represents the functional relationship between ENC and GC3 under mutation pressure rather than natural selection. X-axis represents GC3%, while Y-axis represents ENC. **A** *Babesia* spp., **B** *Cryptosporidium* spp., **C** *Eimeria* spp., **D** *Plasmodium* spp., **E** *Theileria* spp., **F** others (including *Besnoitia besnoiti*, *Cyclospora cayetanensis*, *Gregarina niphandrodes*, *Hammondia hammondi*, *Neospora caninum*, and *Toxoplasma gondii*)

To further confirm the influence of GC3s on the codon bias of Trxs in apicomplexan protozoa, a distribution plot was employed that deviated from the same usage of indistinguishable codons (Fig. 5). In this study, ENC values were used against the GC3, and the standard curve indicates that the functional relationship between ENC and GC3 is influenced by mutation pressure rather than natural selection. If the GC subject of the gene exhibits mutational pressure, all the points in this plot will lie on the expected curve, indicating random codon usage. However, if there was natural selection pressure on the gene, most of the points were below the expected curve and just a few points beyond it (*Babesia bovis*, *B. microti*, *B. ovata*, *Eimeria tenella*, *Plasmodium knowlesi*, *P. malariae*, *G. niphandrodes*). The results showed that all of the points were closed to the standard curve without lying on it, which indicates that mutation pressure is not the only factor that shapes codon bias, and natural selection also plays a key role in codon bias formation.

### Results of correlation analysis in apicomplexan protozoa Trxs

**Fig. 6:**
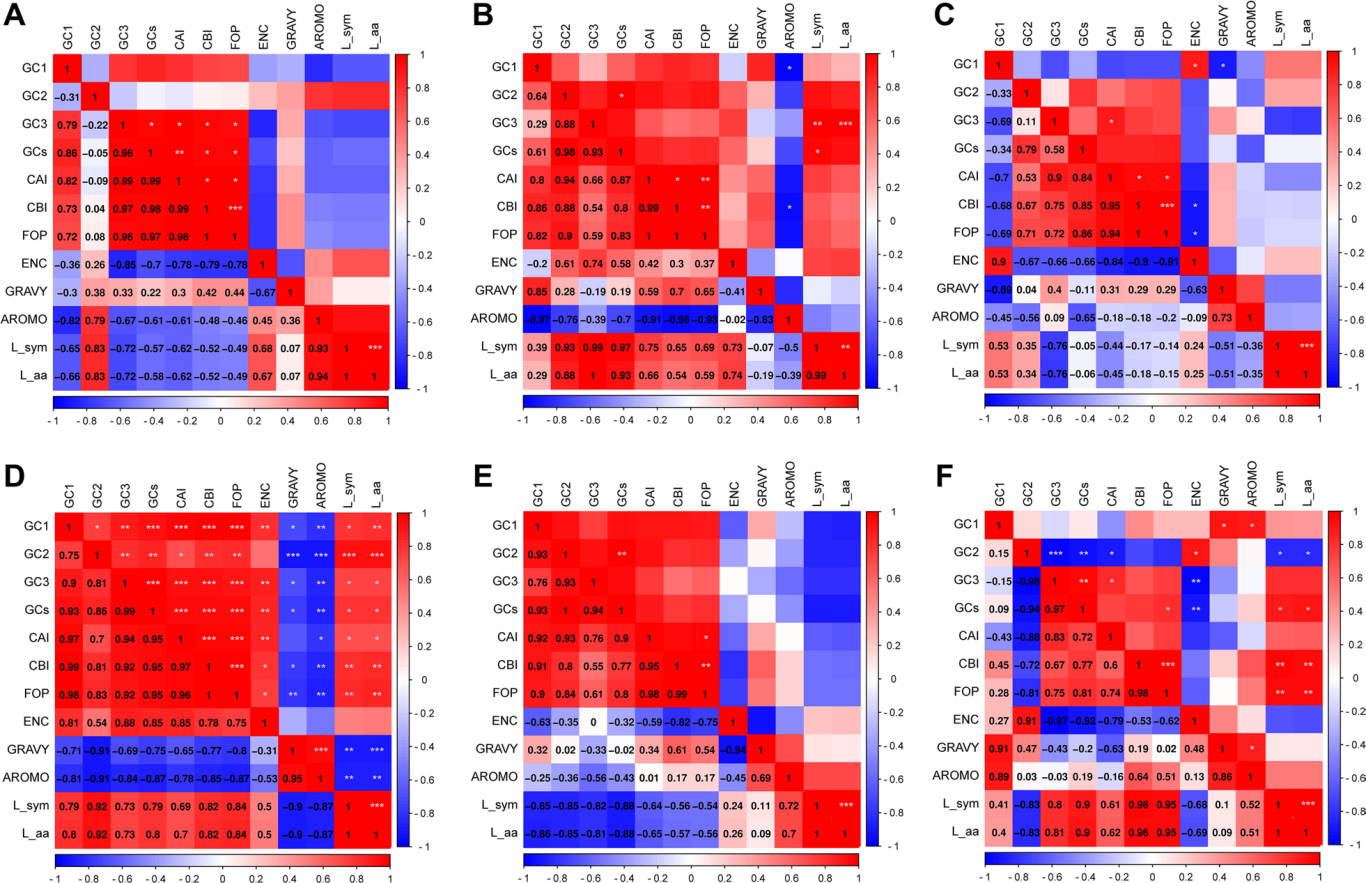
Correlation analysis in apicomplexan protozoa Trxs. The correlations among codon base composition (GC1, GC2, GC3, GCs), codon adaptation index (CAI), codon bias index (CBI), frequency of optimal codons (FOP), effective number of codons (ENC), general average hydropathicity (GRAVY), aromaticity (AROMO), length of synonymous codons (L_sym), and length of amino acids (L_aa) were analyzed in apicomplexan protozoa Trxs, respectively. The color of the color block changes from blue to red, indicating that the correlation is increasing. X-axis represents correlation, while Y-axis represents different apicomplexan protozoa. **A** *Babesia* spp., **B** *Cryptosporidium* spp., **C** *Eimeria* spp., **D** *Plasmodium* spp., **E** *Theileria* spp., **F** others (including *Besnoitia besnoiti*, *Cyclospora cayetanensis*, *Gregarina niphandrodes*, *Hammondia hammondi*, *Neospora caninum*, and *Toxoplasma gondii*). One asterisk (*) indicates a significant correlation among indices at *p* < 0.05; two asterisks (**) indicate the correlation at *p* < 0.01; Three asterisks (***) indicate the correlation at *p* < 0.001

To intuitively display the indices related to the 12 main contributors, correlations of the important indices were calculated to determine the important factors that result in codon bias (Fig. 6). In *Babesia* spp., the values of GC1, GC2, ENC, GRAVY, and AROMO did not correlate with other indices, while GC3 was correlated with GCs, CAI, CBI, and FOP (*p* < 0.05, Fig. 6A). In addition, we did not observe a significant correlation between GC1 and GC2 or GC3 in *Babesia* spp., *Cryptosporidium* spp., *Eimeria* spp., and *Theileria* spp., except *Plamodium* spp. (Fig. 6). CBI value was significantly correlated with the FOP among these apicomplexan protozoa (*p* < 0.01). There was a significant correlation between the ENC and GC1 contents in *Eimeria* spp. and *Plasmodium* spp., which might lead to an assumption about the usage of synonymous codons suffered from natural selection (Fig. 6C, D). Furthermore, only a few indices correlate with *Theileria* spp. (Fig. 6E); however, almost all indices correlate with *Plasmodium* spp. (Fig. 6D), which indicated both mutation pressure and natural selection play a key role in codon bias formation.

### Results of phylogenetic analysis in apicomplexan protozoa Trxs

**Fig. 7:**
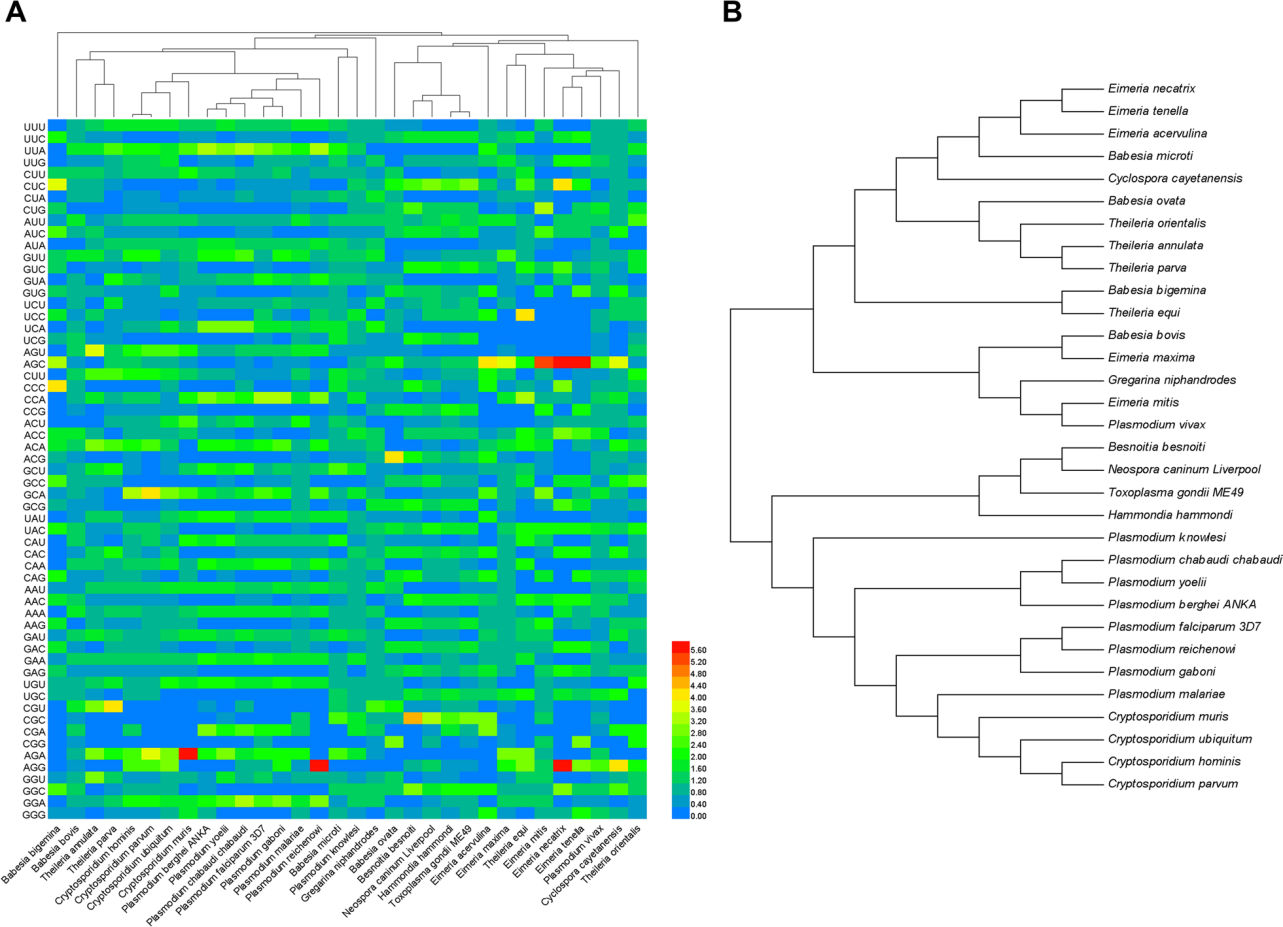
Phylogenetic analysis in apicomplexan protozoa Trxs. The evolution analyses among apicomplexan protozoa Trxs were clustered by relative synonymous codon usage (RSCU) value (**A**, the color of the color block changes from blue to red, indicating that the RSCU values are increasing) and coding sequences (**B**, through the neighbor-joining method), respectively

To assess the consequence of evolutionary procedures on the Trxs in apicomplexan protozoa codon usage patterns, 32 apicomplexan protozoa RSCU values of Trxs were used for the cluster analysis (Fig. 7A). The results showed that all the species are divided into two big clusters at the evolution distance; the *Babesia* spp. and *Theileria* spp. were also separated into different clusters, respectively, while *Plasmodium* spp. and *Cryptosporidium* spp. were in the same cluster. Compared with the phylogenetic relationship based on RSCU, a phylogenetic analysis was used by CDS through the neighbor-joining method (Fig. 7B). Based on CDS phylogenetic analysis, the *Babesia* spp. and *Theileria* spp. were in different evolutionary clades of the same cluster, which is closer to the real evolution.

## Discussion

Across long-term evolution, organisms will eventually develop a specific set of codon usages, which preserves the conveyance of genetic information between nucleotides and amino acids [65, 66]. Nevertheless, disparate genes of the same or distinct species display varying predilections towards codon usage [67]. Consequently, CUB analysis offers valuable insights into the regulatory mechanisms of translation processes and facilitate exogenous gene prediction and optimization for improved expression levels through industrial modification [59, 68]. To date, the characteristics of codon usage for thioredoxin genes of apicomplexan protozoa have not been fully understood.

Trx is a type of redox protein, which plays an important role in metabolic redox regulation, parasite survival, host immune evasion, and the invasion process of apicomplexan protozoa [69–74]. The length and codon base composition of Trxs in apicomplexan protozoa showed large variations, indicating the differentiation of apicomplexan protozoa Trxs. It is reported that the difference in synonymous codons is mainly reflected in the difference in the third codon. In this study, we found that the *Cryptosporidium* spp. and *Plasmodium* spp. tend to end with A/T, which is similar to previous research on *P. falciparum*, *Mycoplasma capricolum*, and *Onchocerca volvulus*, being enriched with A and T. *Eimeria* spp., *Babesia* spp., *H. hammondi*, *N. caninum*, and *T. gondii*, were rich in C3/G3, which proved that one specific gene shows diverse codon usage bias in different species and the results are consistent with the feature of apicomplexan protozoa codon usage in other genes [75, 76]. Most high-frequency Trx codons analyzed by RSCU also show the same tendency of using the third codon in apicomplexan protozoa. In addition, the CAI, CBI, and Fop values of *E. necatrix* were the highest, which indicates a strong codon bias. An ENC value < 35 indicates a strong codon preference [77, 78]. The average ENC of these 32 apicomplexan protozoa was 46.59 in this study; all ENC values except *Eimeria necatrix* (30.77) were > 35, which indicates a weak codon preference among apicomplexan protozoa. Furthermore, we detected the correlations among codon base composition (GC1, GC2, GC3, GCs), CAI, CBI, FOP, ENC, GRAVY, AROMO, L_sym, and L_aa, indicating the influence of base composition and codon usage indices on CUB, which show a significant correlation in *Plasmodium* spp. The neutrality plot analysis, PR2-bias plot analysis, and ENC-GC3 plot analysis further demonstrated that natural selection plays an important role in Trxs of apicomplexan protozoa codon bias. Despite some differences in codon usage indices among apicomplexan protozoa, their common point was that CUB of Trx was affected by strong natural selection.

Apicomplexans are a class of obligate intracellular parasitic protozoa, with a large geographical distribution, which are important pathogens for humans and animals and can cause serious zoonotic diseases such as malaria, toxoplasmosis, and cryptosporidiosis [35, 39–44, 79]. Besides, apicomplexans are believed to have been obtained from Protista, dividing into aconoidasida and conoidasida, including *T. gondii*, *Plasmodium* spp., *Cryptosporidium* spp., *Eimeria* spp., *Babesia* spp., *Theileria* spp., and *N. caninum*. At present, the RSCU clustering and CDS phylogenetic tree are widely used for analyzing the evolutionary relationship of the same gene in different species. These two clustering analysis methods have consistent results in some species, while others differ significantly [2]. In this study, we analyzed the relationship of Trxs in different apicomplexan protozoa based on CDS and RSCU, respectively. Actually, the phylogenetic relationships based on CDS are more reliable, which is different from the RSCU-based relationships, especially for the *Babesia* spp. and *Theileria* spp. However, the genetic relationship between some species was correctly interpreted according to the RSCU value, which was consistent with other studies [60, 80]. The results show that the phylogenetic results based on RSCU can be an important supplement to the phylogenetic results based on the sequence.

## Conclusions

Many factors can result in the CUB of organisms. For the Trxs in apicomplexan protozoa, natural selection is found to dominate the high CUB. We believe that mutation pressure only plays a relatively minor role. Moreover, our study provides new insight into the exploration of setting up new methods for species taxonomy, though a trial still needs to be conducted in the future.

### Supplementary Information

**Additional file 1: Table S1.** Sources of the coding sequence in apicomplexan protozoa Trxs.

**Additional file 2: Table S2.** Codon base composition in apicomplexan protozoa Trxs.

**Additional file 3: Table S3.** Relative synonymous codon usage in apicomplexan protozoa Trxs.

## Data Availability

All data associated with this study are present in the paper or the Additional files. Any other relevant data are available from the corresponding author upon reasonable request.
