# Supplementary material for: Analysis of codon usage bias of thioredoxin in apicomplexan protozoa
Source: Parasit Vectors. 2023 Nov 21;16:431. doi: 10.1186/s13071-023-06002-w (PMC10664530; doi:10.1186/s13071-023-06002-w)
Supplement: Supplementary file 1 — Additional file 1: Table S1. Sources of the coding sequence in apicomplexan protozoa Trxs. [file 13071_2023_6002_MOESM1_ESM.docx]

**Table S1 Sources of coding sequence of Trx genes in apicomplexan protozoa**

| **Species** | **Accession Number** | **Encoding region length** | **Number of encoded amino acids** |
| --- | --- | --- | --- |
| *Babesia bigemina* | XM_012911962.1 | 309 | 103 |
| *Babesia bovis* | XM_001610782.2 | 1116 | 372 |
| *Babesia microti* | XM_021481756.1 | 564 | 188 |
| *Babesia ovata* | XM_029011511.1 | 420 | 140 |
| *Besnoitia besnoiti* | XM_029361110.1 | 1284 | 428 |
| *Cryptosporidium hominis* | XM_663102.1 | 603 | 201 |
| *Cryptosporidium muris* | XM_002139223.1 | 555 | 185 |
| *Cryptosporidium parvum* | XM_626144.1 | 603 | 201 |
| *Cryptosporidium ubiquitum* | XM_029018901.1 | 603 | 201 |
| *Cyclospora cayetanensis* | XM_022731666.1 | 315 | 105 |
| *Eimeria acervulina* | XM_013391257.1 | 306 | 102 |
| *Eimeria maxima* | XM_013477497.1 | 1299 | 433 |
| *Eimeria mitis* | XM_013500698.1 | 915 | 305 |
| *Eimeria necatrix* | XM_013577422.1 | 255 | 85 |
| *Eimeria tenella* | XM_013375329.1 | 309 | 103 |
| *Gregarina niphandrodes* | XM_011134950.1 | 591 | 197 |
| *Hammondia hammondi* | XM_008888728.1 | 1269 | 423 |
| *Neospora caninum Liverpool* | XM_003879921.1 | 1284 | 428 |
| *Plasmodium berghei* ANKA | XM_034565040.1 | 1260 | 420 |
| *Plasmodium chabaudi chabaudi* | XM_740604.1 | 1260 | 420 |
| *Plasmodium falciparum* 3D7 | XM_001347695.1 | 1272 | 424 |
| *Plasmodium gaboni* | XM_018786170.1 | 1272 | 424 |
| *Plasmodium knowlesi* | XM_002259008.1 | 1281 | 427 |
| *Plasmodium malariae* | XM_029004819.1 | 1260 | 420 |
| *Plasmodium reichenowi* | XM_020114914.1 | 987 | 329 |
| *Plasmodium vivax* | XM_024731245.1 | 1665 | 555 |
| *Plasmodium yoelii* | XM_718815.1 | 1260 | 420 |
| *Theileria annulata* | XM_947576.1 | 447 | 149 |
| *Theileria equi* | XM_004830685.1 | 315 | 105 |
| *Theileria orientalis* | XM_009691758.1 | 420 | 140 |
| *Theileria parva* | XM_759511.1 | 672 | 224 |
| *Toxoplasma gondii* ME49 | XM_018779488.1 | 1272 | 424 |
