# Supplementary material for: Analysis of codon usage bias of thioredoxin in apicomplexan protozoa
Source: Parasit Vectors. 2023 Nov 21;16:431. doi: 10.1186/s13071-023-06002-w (PMC10664530; doi:10.1186/s13071-023-06002-w)
Supplement: Supplementary file 2 — Additional file 2: Table S2. Codon base composition in apicomplexan protozoa Trxs. [file 13071_2023_6002_MOESM2_ESM.docx]

**Table S2 Codon base composition of Trx genes in apicomplexan protozoa**

| **Species** | **A3** | **T3** | **C3** | **G3** | **GC1** | **GC2** | **GC3** | **GCs** | **ATs** |
| --- | --- | --- | --- | --- | --- | --- | --- | --- | --- |
| *Babesia bigemina* | 0.0779 | 0.1358 | 0.7037 | 0.3571 | 0.5192 | 0.3558 | 0.8269 | 0.5673 | 0.4327 |
| *Babesia bovis* | 0.3386 | 0.4013 | 0.3229 | 0.2028 | 0.4053 | 0.4709 | 0.4029 | 0.4264 | 0.5736 |
| *Babesia microti* | 0.3864 | 0.4762 | 0.2041 | 0.2743 | 0.4127 | 0.3122 | 0.3545 | 0.3598 | 0.6402 |
| *Babesia ovata* | 0.1481 | 0.2358 | 0.4528 | 0.4706 | 0.6312 | 0.3546 | 0.7092 | 0.5650 | 0.435 |
| *Besnoitia besnoiti* | 0.0669 | 0.1206 | 0.5587 | 0.5538 | 0.5338 | 0.3473 | 0.8578 | 0.5796 | 0.4204 |
| *Cryptosporidium hominis* | 0.5000 | 0.4067 | 0.1933 | 0.2348 | 0.4010 | 0.2822 | 0.3119 | 0.3317 | 0.6683 |
| *Cryptosporidium muris* | 0.4597 | 0.5652 | 0.1449 | 0.2037 | 0.3925 | 0.2527 | 0.2688 | 0.3047 | 0.6953 |
| *Cryptosporidium parvum* | 0.4967 | 0.4161 | 0.1879 | 0.2331 | 0.4059 | 0.2772 | 0.3119 | 0.3317 | 0.6683 |
| *Cryptosporidium ubiquitum* | 0.4600 | 0.4792 | 0.1528 | 0.2734 | 0.3861 | 0.2673 | 0.3119 | 0.3218 | 0.6782 |
| *Cyclospora cayetanensis* | 0.2468 | 0.2692 | 0.3974 | 0.4085 | 0.4811 | 0.3774 | 0.6132 | 0.4906 | 0.5094 |
| *Eimeria acervulina* | 0.2838 | 0.2535 | 0.3803 | 0.4348 | 0.4854 | 0.3204 | 0.6117 | 0.4725 | 0.5275 |
| *Eimeria maxima* | 0.3333 | 0.3077 | 0.3462 | 0.2741 | 0.5253 | 0.4124 | 0.5046 | 0.4808 | 0.5192 |
| *Eimeria mitis* | 0.2355 | 0.1500 | 0.4808 | 0.2925 | 0.5033 | 0.6275 | 0.6699 | 0.6002 | 0.3998 |
| *Eimeria necatrix* | 0.1071 | 0.0986 | 0.7887 | 0.2400 | 0.3140 | 0.5233 | 0.8488 | 0.5620 | 0.438 |
| *Eimeria tenella* | 0.1757 | 0.1096 | 0.5479 | 0.5441 | 0.5096 | 0.2885 | 0.7885 | 0.5288 | 0.4712 |
| *Gregarina niphandrodes* | 0.3667 | 0.3232 | 0.2927 | 0.2552 | 0.5707 | 0.4040 | 0.4545 | 0.4764 | 0.5236 |
| *Hammondia hammondi* | 0.1492 | 0.1975 | 0.5154 | 0.4323 | 0.5307 | 0.3538 | 0.7358 | 0.5401 | 0.4599 |
| *Neospora caninum Liverpool* | 0.1375 | 0.1576 | 0.5697 | 0.4323 | 0.5431 | 0.3473 | 0.7739 | 0.5548 | 0.4452 |
| *Plasmodium berghei* ANKA | 0.6893 | 0.5285 | 0.1013 | 0.0579 | 0.3444 | 0.2399 | 0.1425 | 0.2423 | 0.7577 |
| *Plasmodium chabaudi chabaudi* | 0.6643 | 0.4953 | 0.1577 | 0.0533 | 0.3658 | 0.2518 | 0.1829 | 0.2668 | 0.7332 |
| *Plasmodium falciparum* 3D7 | 0.6563 | 0.4503 | 0.1739 | 0.0906 | 0.3459 | 0.2471 | 0.2118 | 0.2682 | 0.7318 |
| *Plasmodium gaboni* | 0.6551 | 0.5093 | 0.1235 | 0.0797 | 0.3388 | 0.2471 | 0.1671 | 0.2510 | 0.749 |
| *Plasmodium knowlesi* | 0.4406 | 0.3754 | 0.3477 | 0.2016 | 0.4182 | 0.2477 | 0.4182 | 0.3614 | 0.6386 |
| *Plasmodium malariae* | 0.5159 | 0.5046 | 0.1517 | 0.1928 | 0.3539 | 0.2589 | 0.2637 | 0.2922 | 0.7078 |
| *Plasmodium reichenowi* | 0.6712 | 0.4862 | 0.1344 | 0.0942 | 0.3131 | 0.2310 | 0.1793 | 0.2411 | 0.7589 |
| *Plasmodium vivax* | 0.3455 | 0.262 | 0.3966 | 0.3671 | 0.4314 | 0.3191 | 0.5597 | 0.4367 | 0.5633 |
| *Plasmodium yoelii* | 0.6915 | 0.5489 | 0.0946 | 0.0327 | 0.3492 | 0.2470 | 0.1211 | 0.2391 | 0.7609 |
| *Theileria annulata* | 0.4074 | 0.4667 | 0.2333 | 0.1895 | 0.4467 | 0.2733 | 0.3267 | 0.3489 | 0.6511 |
| *Theileria equi* | 0.378 | 0.2317 | 0.5366 | 0.1139 | 0.6226 | 0.3585 | 0.5283 | 0.5031 | 0.4969 |
| *Theileria orientalis* | 0.2039 | 0.4528 | 0.283 | 0.3830 | 0.4468 | 0.3121 | 0.5035 | 0.4208 | 0.5792 |
| *Theileria parva* | 0.4710 | 0.4615 | 0.2088 | 0.1894 | 0.3755 | 0.2620 | 0.3013 | 0.3130 | 0.687 |
| *Toxoplasma gondii* ME49 | 0.1302 | 0.1698 | 0.537 | 0.4603 | 0.5435 | 0.3506 | 0.7718 | 0.5553 | 0.4447 |
